# Drug Discovery for Duchenne Muscular Dystrophy via Utrophin Promoter Activation Screening

**DOI:** 10.1371/journal.pone.0026169

**Published:** 2011-10-20

**Authors:** Catherine Moorwood, Olga Lozynska, Neha Suri, Andrew D. Napper, Scott L. Diamond, Tejvir S. Khurana

**Affiliations:** 1 Department of Physiology and Pennsylvania Muscle Institute, University of Pennsylvania School of Medicine, Philadelphia, Pennsylvania, United States of America; 2 Penn Center for Molecular Discovery, Institute for Medicine and Engineering, University of Pennsylvania, Philadelphia, Pennsylvania, United States of America; University of Helsinki, Finland

## Abstract

**Background:**

Duchenne muscular dystrophy (DMD) is a devastating muscle wasting disease caused by mutations in dystrophin, a muscle cytoskeletal protein. Utrophin is a homologue of dystrophin that can functionally compensate for its absence when expressed at increased levels in the myofibre, as shown by studies in dystrophin-deficient mice. Utrophin upregulation is therefore a promising therapeutic approach for DMD. The use of a small, drug-like molecule to achieve utrophin upregulation offers obvious advantages in terms of delivery and bioavailability. Furthermore, much of the time and expense involved in the development of a new drug can be eliminated by screening molecules that are already approved for clinical use.

**Methodology/Principal Findings:**

We developed and validated a cell-based, high-throughput screening assay for utrophin promoter activation, and used it to screen the Prestwick Chemical Library of marketed drugs and natural compounds. Initial screening produced 20 hit molecules, 14 of which exhibited dose-dependent activation of the utrophin promoter and were confirmed as hits. Independent validation demonstrated that one of these compounds, nabumetone, is able to upregulate endogenous utrophin mRNA and protein, in C2C12 muscle cells.

**Conclusions/Significance:**

We have developed a cell-based, high-throughput screening utrophin promoter assay. Using this assay, we identified and validated a utrophin promoter-activating drug, nabumetone, for which pharmacokinetics and safety in humans are already well described, and which represents a lead compound for utrophin upregulation as a therapy for DMD.

## Introduction

Duchenne muscular dystrophy (DMD) is a devastating X-linked muscle wasting disease, caused by mutations in the dystrophin gene [1], [2]. Dystrophin provides structural integrity to skeletal and cardiac muscle by linking the subsarcolemmal actin cytoskeleton to the extracellular matrix, via the dystrophin associated protein complex (DAPC). In the absence of dystrophin, the entire DAPC is lost from the sarcolemma [3]. Muscles are unable to transmit force efficiently and become susceptible to damage during contraction, leading to cycles of degeneration and regeneration. Eventually, regeneration fails and muscle fibres are replaced by fatty and fibrous tissue [2]. Calcium misregulation and chronic inflammation are also thought to contribute to the phenotype [4], [5], [6]. For patients, DMD leads to progressive muscle weakness, dependence on a wheelchair, respiratory and cardiac complications and a shortened lifespan [7], [8]. There is currently no effective treatment available.

Utrophin is an autosomal homologue of dystrophin that can also bind to proteins of the DAPC [9], [10], [11], [12]. Dystrophin and utrophin share 74% similarity at the amino acid level and have very similar domain structures [12], [13]. Utrophin is expressed in place of dystrophin in foetal muscle, but in adult myofibres is confined to the neuromuscular and myotendinous junctions. Utrophin is also expressed in other tissues including lung, kidney and liver [9], [14]. There are two isoforms of utrophin, A and B, that are transcribed from different promoters [15]. Utrophin A is the predominant isoform in the myofibre [16]. Studies in *mdx* mice, a model for DMD, have shown that utrophin, when overexpressed in myofibres by viral vector-mediated delivery or by transgenic means, can compensate for the absence of dystrophin, restoring normal muscle function [17], [18]. It is also worth noting that, because utrophin is expressed in foetal muscle and in various non-muscle tissues in the adult [9], [10], its overexpression in the muscles of people with DMD is unlikely to provoke an immune response. Utrophin upregulation is therefore an attractive therapeutic approach for DMD. Preclinical investigations of utrophin-upregulating treatments, such as heregulin, L-arginine, viral delivery of an artificial transcription factor targeting the utrophin promoter or direct administration of a TAT-tagged ‘microutrophin’ protein have yielded promising improvements in the *mdx* phenotype [19], [20], [21], [22], [23]. However, no utrophin upregulation therapy is yet available for clinical use in DMD patients.

In contrast to protein or virus-based therapeutics, a small-compound drug for utrophin upregulation would avoid potential obstacles in terms of delivery, safety and regulatory body approval. The process of drug discovery, from high-throughput screening through lead optimisation, in vivo studies, clinical trials and eventual approval for patient use, is protracted and expensive, with high failure rates [24], [25], [26]. An accelerated passage to the clinic and an improved chance of success could be achieved by screening compounds that are already approved for other indications [27], [28], [29]. Indeed, this approach was successful in identifying β-lactam antibiotics as potential new drugs for amyotrophic lateral sclerosis [30]. With this in mind, we developed a cell-based, high-throughput assay for utrophin A promoter activation, and used it to screen the Prestwick Chemical Library, which comprises 90% approved drugs and 10% natural compounds. Initial screening generated 20 hits out of 1120 compounds (1.8%). Further testing for dose-dependent utrophin promoter activation confirmed 14 molecules as hits, one of which, nabumetone, was shown to upregulate endogenous utrophin A mRNA and protein, in independent validation experiments using the C2C12 muscle cell line. This drug, for which pharmacokinetics, bioavailability and safety in humans are already well described, represents a potential therapeutic candidate for DMD.

## Methods

### Chemicals

Trichostatin A (TSA) was purchased from Wako; a stock solution of 0.1 mg/ml (331 µM) was prepared by dissolving in methanol. Heregulin-β1 EGF domain was purchased from R&D systems; a 1.25 µM stock solution was prepared by dissolving in sterile PBS supplemented with 0.1% bovine serum albumin. L-arginine was purchased from Sigma; a 100 mg/ml (574 mM) stock solution was made by dissolving in sterile water. Okadaic acid was purchased from Sigma; a stock solution of 20 mM was made by dissolving in DMSO. The Prestwick Chemical Library was purchased from Prestwick Chemical. The 1120 compounds were supplied at 2 mg/ml in DMSO, in 96-well format. For screening, the library was reformatted to 384-well format.

### Cell Culture

C2C12 cells (purchased from ATCC) were cultured in high glucose DMEM with 10% foetal bovine serum (FBS), 2 mM L-glutamine, 100 U/ml penicillin and 0.1 mg/ml streptomycin. Cell culture reagents were purchased from Gibco. For the C2C12utrn stable cell line, 250 mg/ml hygromycin B (Roche) was added to the media. Cells were plated at 1200 cells/well in 384-well plates, 3000 cells/well in 96-well plates or 60 000 cells/well in 6-well plates. For 384-well plates, cells were seeded using a Matrix Wellmate (Thermo Scientific).

### Luciferase Assays

Luciferase assays were done using the BrightGlo assay (Promega), following the manufacturer's instructions. For assays done in 96-well format, luminescence was recorded using a Luminoskan Ascent luminometer (Thermo Labsystems). For assays done in 384-well format, luminescence was recorded using an Envision plate reader (Perkin Elmer).

### Cell Interference Assay

QuantiLum recombinant luciferase (Promega) diluted in C2C12 media was added to 96-well plates with or without C2C12 cells at concentrations of 10^−9^ to 10^−14^ M (50 µl/well). Luciferase activity was assayed as described above. Statistical analysis was done by two-way ANOVA, using GraphPad Prism 4 (GraphPad Software, Inc).

### Generation of pGL4:14/utrnAprom Construct

The 2.3 kb human utrophin A promoter fragment was amplified by PCR from CHORI BAC clone PR1-91J24 (EMBL accession no. AL024474), using the primers 5′-TCAAACACTCCAATGTGGCCTTATTATCTA-3′ and 5′-TAAAGCTTGGAGAAGCAGACACGAAC-3′. The PCR product was TA-cloned into the pCR2.1-TOPO vector (Invitrogen) and completely sequenced before subcloning into the multiple cloning site of pGL4:14 (Promega) using the restriction enzymes *Kpn*I and *Eco*RI, to generate the construct pGL4:14/utrnAprom.

### Generation of C2C12utrn Stable Cell Line

C2C12 cells were transfected with the pGL4:14/utrnAprom construct using Lipofectamine2000 (Invitrogen), following the manufacturer's instructions. After 24 hours, media was changed and after 48 hours, cells were trypsinised and re-plated in media supplemented with 250 mg/ml hygromycin B. Resistant colonies were removed using filter paper and cells were re-plated in 96-well plates with sequential dilution so that cell numbers decreased to 0 across multiple wells. From the wells with the lowest starting cell number in which cells survived and multiplied, cells were harvested and subjected to a second round of plating with sequential dilution. The wells with the lowest starting cell number were again selected and clones were expanded into 24-well plates.

### Validation of C2C12utrn Stable Cell Line

C2C12utrn clones were first validated using the BrightGlo luciferase assay (Promega). From the four clones with the highest luciferase activity, genomic DNA was isolated using the ArchivePure DNA Cell/Tissue Kit (5Prime, Inc). For PCR validation, the primers 5′-ACTCTGGAGCGCGCGCCCCA-3′ and 5′-CGCCTCTGCAGCGCTCCGGCTC-3′, which bind specifically to the utrophin A promoter, were used for amplification from 300 ng of genomic DNA. The validated clone with the highest luciferase activity was used for all subsequent experiments.

### DMSO Toxicity and Positive Control Evaluation

For evaluation of sensitivity to DMSO, C2C12utrn cells were seeded in 96-well plates and treated with DMSO (Sigma) at final concentrations of 0–0.5%. Luciferase activity was assayed after 48 hours, as described above.

For evaluation of potential positive controls, C2C12utrn cells were seeded in 96-well plates 24 hours prior to compound treatment. Cells were exposed to L-arginine (2 mM), TSA (50 nM), okadaic acid (50 nM) or heregulin (2 nM) for 24 or 48 hours before assaying luciferase activity. Differences between treatments were tested by one-way ANOVA using Statview software (SAS Institute, Inc). Statistical robustness was assessed by Z-factor, defined as 1−((3×(SD control+SD treated))/(mean treated−mean control)) and percentage covariance (% CV).

For generation of the TSA dose-response curve, C2C12utrn cells were plated in 384-well plates 24 hours prior to treatment with TSA. Five dilutions of TSA from 0.3 to 5000 µM were prepared in 100% DMSO. TSA was added to cells at final concentrations ranging from 0.3 to 5000 nM using an Evolution P^3^ robot liquid handling system (Perkin Elmer). The final DMSO concentration was 0.1%. Luciferase activity was assayed after 24 hours. Sigmoidal dose-response curve-fitting and EC50 calculation was done by non-linear regression using GraphPad Prism 4 (GraphPad Software, Inc).

### Initial Screen

C2C12utrn cells were plated in 384-well plates 24 hours prior to treatment with compounds. Compounds were added at a final concentration of 2 µg/ml (approximately 5 µM based on an average molecular weight of 400) using the Evolution P^3^ robot liquid handling system (Perkin Elmer). The final DMSO concentration was 0.1%. Negative controls were treated with 0.1% DMSO only. Quality control (QC) plates were treated with 5 nM TSA. After 24 hours compound exposure, luciferase activity was assayed as described above. QC plates were run at the beginning and end of the assay. An algorithm for cross-talk correction was applied. Data analysis was done using ActivityBase (IDBS). The threshold for hits was set at 20% upregulation (approximately 3 times the % CV of the negative controls).

### Hit Confirmation and Dose Optimisation

For hit confirmation (dose-response I), C2C12utrn cells were treated with each of the hits from the initial screen at 15 concentrations from 0.5 ng/ml to 8 µg/ml (approximately 1.6 nM to 25 µM based on an average molecular weight for these 20 compounds of 316), to generate dose-response curves. The final DMSO concentration remained constant at 0.4%. Otherwise, the protocol was as described for the initial screen. For dose optimisation (dose-response II), compounds were obtained in greater quantity and dissolved in DMSO at 100 or 200 mM. C2C12utrn cells were treated at 16 concentrations from 3.1 nM to 200 µM. The final DMSO concentration remained constant at 0.1%. The protocol was otherwise as for the initial screen.

### Validation by Quantitative Real-Time PCR

C2C12 cells were plated in 6-well plates 24 hours prior to treatment with nabumetone (25 µM). The final DMSO concentration was 0.05%. Control cells were treated with 0.05% DMSO only. After 24 hours compound treatment, cells were lysed and RNA isolated using an RNeasy kit (Qiagen), following the manufacturer's protocol. RNA was reverse-transcribed using a Superscript II First-Strand Synthesis kit (Invitrogen). A custom TaqMan quantitative real-time PCR (qRT-PCR) assay for utrophin A [31] was performed using 500 nM each of primers 5′-ACGAATTCAGTGACATCATTAAGTCC-3′ and 5′-ATCCATTTGGTAAAGGTTTTCTTCTG-3′ and 250 nM of FAM-labelled MGB probe with sequence ATCATTGTGTTCATCAGATC, with 8 ng cDNA in a reaction volume of 25 µl. As an endogenous control, 18S rRNA was amplified using pre-mixed reagents from Applied Biosystems (Eukaryotic 18S rRNA Endogenous Control (VIC/MGB probe, primer limited)), with 0.8 ng cDNA in a reaction volume of 50 µl. Other reaction components were provided by Applied Biosystems TaqMan Universal Mastermix. TaqMan qRT-PCR reactions were carried out in 96-well plates using a 7300 Real-Time PCR System (Applied Biosystems) and default thermocycler program. Analysis was done using the ΔΔCt method, having previously validated the equal efficiencies of the two primer sets. Statistical analysis of multiple independent experiments was done by one-way ANOVA using Statview software (SAS Institute, Inc).

### Validation by Western Blotting

Cells were plated in 60 mm dishes such that confluence was approximately 25% the following day, at which point they were treated with 25 µM nabumetone or vehicle (DMSO) only (0.1%) for 4 days. After 2 days, cells were passaged and re-seeded in fresh media with nabumetone or DMSO. After 4 days, cells were trypsinised and resuspended in 300 µl TNEC lysis buffer (1.5 mM Tris-HCl pH 8, 2.15 mM NaCl, 3.1% Igepal CA630, 4.2 mM EDTA with Complete protease inhibitors (Roche)). Lysates were incubated on ice for 20 minutes, centrifuged at maximum speed for 10 minutes in a benchtop centrifuge at 4°C and supernatants removed and stored at −20°C. The DC protein assay (Bio-Rad) was used to determine total protein concentration. For Western blotting, lysates containing 30 µg protein were combined with LDS sample buffer and NuPAGE reducing agent and heated to 99°C for 5 minutes, then separated on 3–8% Tris-Acetate gels (Invitrogen) with TA running buffer for 2 hours 15 minutes at 80 V. Proteins were transferred to PVDF membranes for 2 hours at 80 V in ice-cooled transfer buffer (25 mM Tris pH 8.3, 192 mM glycine, 20% methanol, 0.05% sodium dodecyl sulphate). Membranes were blocked overnight at 4°C in 5% non-fat milk in TBS (50 mM Tris pH 7.5, 150 mM NaCl), then probed for utrophin with mouse monoclonal anti-utrophin antibody mancho 3 clone 8A4 (developed by Glenn E. Morris and obtained from the Developmental Studies Hybridoma Bank developed under the auspices of the NICHD and maintained by The University of Iowa Department of Biology) diluted 1∶20 in 5% non-fat milk in TBST (TBS with 0.05% Tween 20), for 1 hour at room temperature. Blots were washed in 3 changes of TBST for 10 minutes each, then incubated with HRP-conjugated goat-anti-mouse IgG (Jackson ImmunoResearch), diluted 1∶4000 in 5% non-fat milk in TBS, for 1 hour. TBST washes were repeated, then bands were visualised using SuperSignal West Pico Chemiluminescent Substrate (Thermo Scientific) and images obtained using an LAS-3000 Imager (Fujifilm). Band densities were quantified using ImageJ (http://rsbweb.nih.gov/ij/index.html). Statistical analysis of multiple independent experiments was done by Student's T test using GraphPad Prism 5 (GraphPad Software, Inc).

## Results

### Generation and Validation of C2C12utrn Stable Cell Line

The C2C12 mouse muscle cell line was selected for generation of a stable cell line containing the human utrophin A promoter linked to a luciferase reporter. Before making the cell line, luciferase assays were performed with a known range of concentrations of recombinant luciferase, in the presence or absence of C2C12 cells. The presence of C2C12 cells had no significant effect on the measured luciferase activity (Fig. S1A). C2C12 cells were then transfected with a construct containing a 2.3 kb utrophin A promoter region linked to a luciferase reporter gene. Hygromycin (250 mg/ml) was used to select stably transfected cells, and resistant colonies were subjected to 2 rounds of sub-cloning to obtain homogenous lines. Clones were validated using both a luciferase assay and PCR with utrophin A promoter-specific primers and genomic DNA as template (data not shown). The clone with the greatest luciferase activity (named C2C12utrn) was selected for development of a cell-based utrophin promoter activation assay.

### Utrophin Promoter Activation Assay Development

C2C12utrn cell number was optimised to 3000 cells per well, which resulted in 70% confluence at the time of luciferase quantification. DMSO is typically used as a solvent when screening chemical libraries. To determine the tolerance of the C2C12utrn cell line to DMSO, cells were treated with a range of DMSO concentrations from 0–0.5% and their luciferase activity was assayed. Concentrations of DMSO up to 0.2% had little effect on measured luciferase activity. Above 0.2% DMSO, luciferase activity declined but at 0.5% DMSO the activity was still 75% that of cells without DMSO (Fig. S1B).

**Figure 1:**
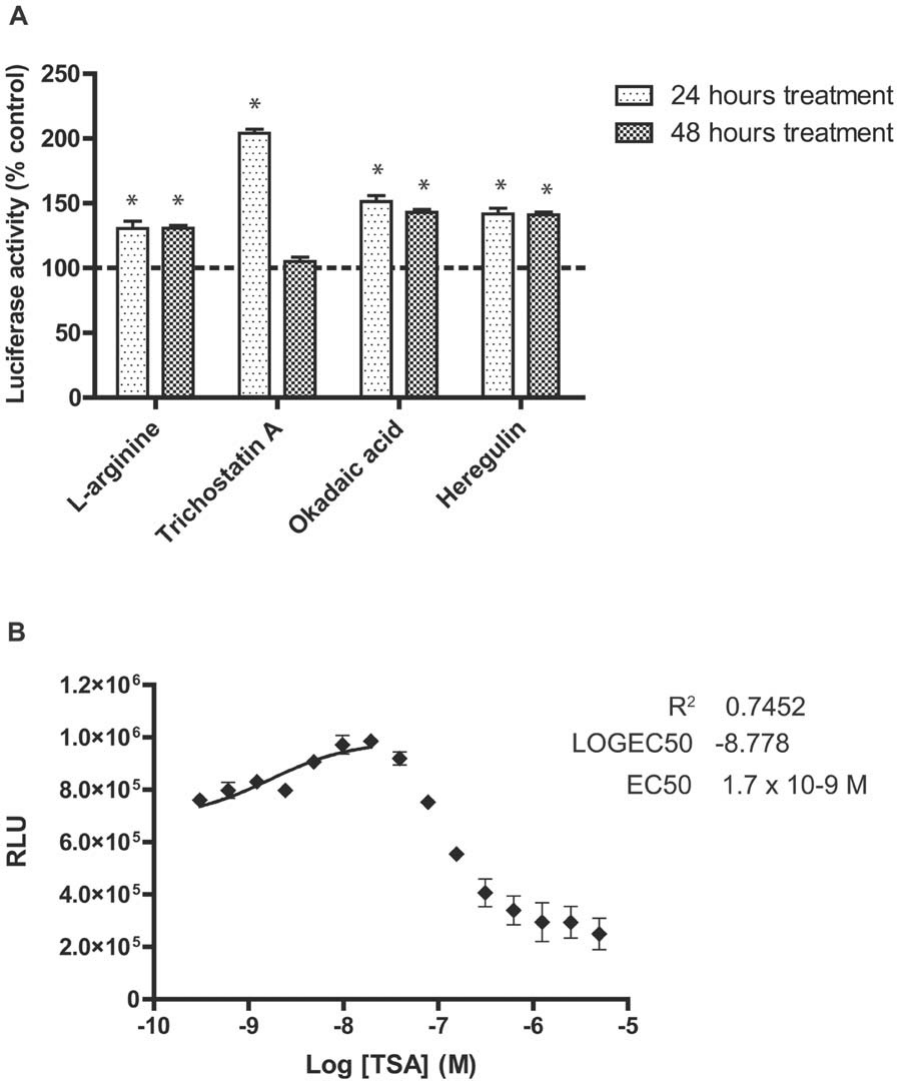
Evaluation of positive controls and incubation times. **A.** C2C12utrn cells were treated for 24 or 48 hours with four compounds known to upregulate utrophin, in 96-well format. The greatest upregulation (2-fold) was seen with trichostatin A (TSA) after 24 hours treatment. Bars represent means ± standard deviation. Dotted line represents control luciferase activity. * Different from untreated C2C12utrn cells (p<0.0001). **B.** A dose-response curve was generated for treatment of C2C12utrn cells with TSA in 384-well format. The greatest response was seen at 20 nM and the EC50 was 1.7 nM. Error bars represent standard deviation. RLU, relative luminescence units.

To find a positive control to assist in assay optimisation and to examine the effects of different compound exposure times, C2C12utrn cells were treated with four compounds previously demonstrated to upregulate utrophin: L-arginine (2 mM) [32], okadaic acid (50 nM) [33], heregulin (2 nM) [34] and trichostatin A (TSA; 50 nM) (Bogdanovich *et al.*, manuscript in preparation), for 24 or 48 hours, before assaying for luciferase activity. Incubation with TSA for 24 hours gave the greatest upregulation of luciferase activity. For the other positive controls, the treatment time did not affect the degree of promoter activation (Fig. 1A). To determine the statistical robustness of the observed upregulation, Z-factors were calculated for each positive control. Only TSA, with 24 hours incubation, had a Z-factor indicative of suitability for high-throughput screening (0.6; acceptable range 0.5–1) [35]. Additionally, the percentage covariance (% CV) was under 10% and lower than for most other treatments (Table 1). Based on these results, a compound exposure time of 24 hours was chosen for the assay and TSA was selected as a positive control for further assay development.

**Table 1:** Determination of Z-factor and % CV for positive controls.

Positive control	24 h treatment		48 h treatment	
	Z-factor	% CV	Z-factor	% CV
L-arginine	−1	10	−0.3	7.6
Trichostatin A	0.6	3.6	−8	7.8
Okadaic acid	−0.1	7.5	0.06	4.1
Heregulin	−0.3	7.5	0.08	3.5

C2C12utrn cells cultured in 96-well plates were exposed to L-arginine (2 mM), TSA (50 nM), okadaic acid (50 nM) or heregulin (2 nM) for 24 or 48 hours before assaying luciferase activity. Statistical robustness was assessed by calculating the Z-factor and percentage covariance (% CV). Z-factors between 0.5 and 1 predict good performance in high-throughput screening.

To further characterise the effect of TSA on C2C12utrn luciferase activity and to confirm that the assay would translate to high-throughput format, a dose-response curve was constructed for TSA in 384-well format using concentrations from 0.3 to 5000 nM. Non-linear regression was used to fit a sigmoidal dose-response curve. From this, the EC50 was calculated to be 1.7 nM. Peak luciferase activity occurred at 20 nM, above which the activity declined, presumably due to cellular toxicity (Fig. 1B). Cells are typically more susceptible to toxic effects in smaller sized plate wells (unpublished observations). Z-factors and % CVs calculated for TSA in 384-well format were similar to those obtained in 96-well format (Table 2), confirming that the assay would perform robustly in high-throughput screening.

**Table 2:** Determination of Z-factor and % CV for TSA in 384-well format.

TSA concentration (nM)	Z-factor	% CV
5	0.7	1.3
20	0.6	3.3

C2C12utrn cells cultured in 384-well plates were exposed to TSA at a range of concentrations. Peak luciferase activity occurred at 20 nM. To determine statistical robustness in 384-well format, the Z-factor and percentage covariance (% CV) were calculated at 5 and 20 nM. Z-factors between 0.5 and 1 predict good performance in high-throughput screening.

### Primary Screen of Prestwick Chemical Library

**Figure 2:**
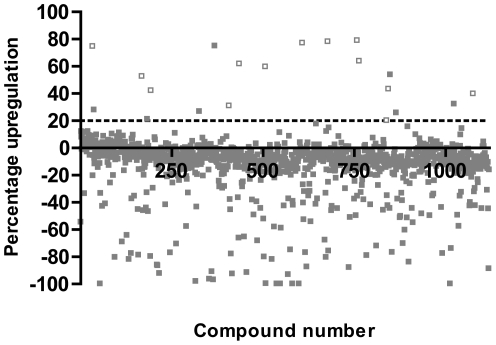
Initial screen of Prestwick Chemical Library. The utrophin promoter activation assay was used to screen the Prestwick Chemical Library of approved drugs and natural products. Using a threshold of 20% upregulation (dotted line), 20 out of 1120 compounds were identified as hits (1.8%). Compounds confirmed as hits after dose-response testing are represented by open squares.

The utrophin promoter activation assay was used to screen the Prestwick Chemical Library of approved drugs and natural compounds. The compounds were screened at 2 µg/ml (approximately 5 µM based on an average molecular weight of 400). The control % CVs for the four plates were between 5.3 and 6.8%. The threshold for hits was set at 20% upregulation (approximately 3 times the % CV of the controls, estimated to be low enough to capture all true positive hits). Out of 1120 compounds, 20 hits were obtained (1.8% of the library) with upregulation up to 80% (1.8-fold; Fig. 2 and Table 3).

**Table 3:** Summary of compound activity.

Compound	Classification	Molecular Weight	Initial Screen(2 µg/ml)	Dose-Response I(to 8 µg/ml)		Dose-Response II(to 200 µM)	
			Fold-change	Concentration†(µg/ml)	Maximum fold-change	Concentration†(µM)	Maximum fold-change
Nabumetone	Approved drug	228	1.8	8	1.6	25	2.6
Chrysin	Natural	254	1.8	0.5	1.5	0.391	1.4
Piperine	Natural	285	1.8	8	1.6	3.13	1.3
Apigenin	Natural	270	1.7	1	1.5	0.781	1.5
Riluzole HCl	Approved drug	234	1.7	1	1.6	25	2.0
Phenazopyridine HCl	Approved drug	213	1.6	4	1.6	3.13	2.9
Resveratrol	Natural	228	1.6	1	1.6	6.25	2.9
Tiabendazole	Approved drug	201	1.6	4	1.9	12.5	2.2
Hesperetin	Natural	302	1.5	8	1.9	100	2.8
Leflunomide	Approved drug	270	1.4	1	1.4	12.5	1.8
Kawain	Natural	230	1.4	4	1.4	100	2.5
Kaempferol	Natural	286	1.4	8	1.8	not tested‡	
Clorgyline HCl	Approved drug	272	1.3	4	1.2	25	3.5
Equilin	Component*	268	1.2	8	1.6	100	2.4

*Component of approved drug.

† Concentration at which maximum fold-change was obtained.

‡ For technical reasons kaempferol was not tested to higher concentrations.

HCl, hydrochloride.

The 14 confirmed hits are presented with a summary of the fold-change (upregulation) in luciferase activity produced in each stage of testing with the assay, as well as concentrations giving optimum activity. HCl, hydrochloride.

Supporting Information Legend.

**Figure 3:**
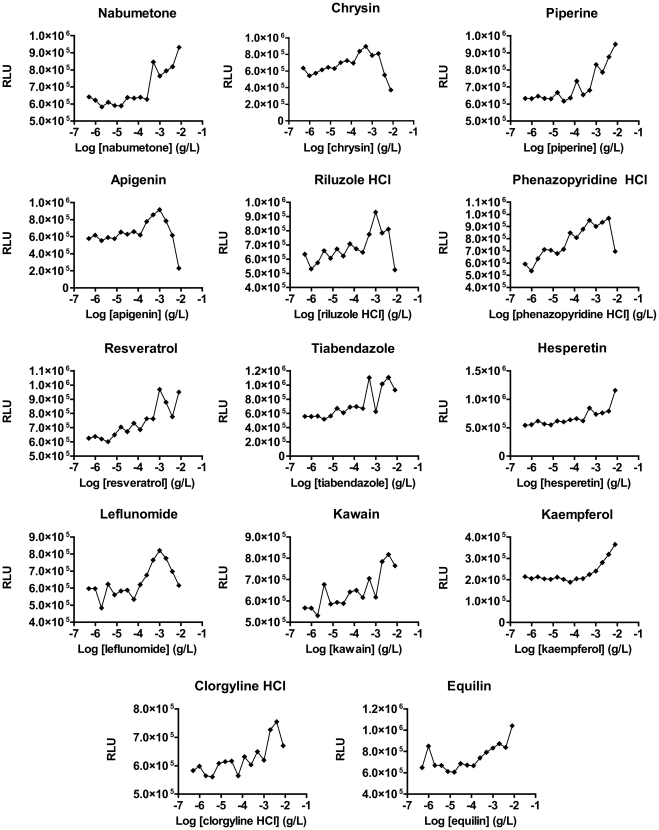
Hit confirmation: dose-response I. Dose-response curves were generated in 384-well format for the 20 compounds identified in the initial screen, using concentrations from 0.5 ng/ml to 8 µg/ml. Fourteen molecules (nabumetone, chrysin, piperine, apigenin, riluzole HCl, phenazopyridine HCl, resveratrol, tiabendazole, hesperetin, leflunomide, kawain, kaempferol, clorgyline HCl and equilin) showed dose-dependent activity and were confirmed as hits. HCl, hydrochloride. RLU, relative luminescence units.

To confirm these hits, high-throughput dose-response curves were generated, using 15 concentrations from 0.5 ng/ml to 8 µg/ml (approximately 1.6 nM to 25 µM based on an average molecular weight for these 20 compounds of 316). Of the 20 initial compounds, 14 showed dose-dependent activation of the utrophin A promoter, confirming them as hits (dose-response I; Fig. 3 and Table 3). These included 7 approved drugs and 7 natural compounds. Maximum fold-changes in utrophin A promoter activity obtained during dose-response testing ranged from 1.2 to 1.9 (Table 3). Dose-response curves were also generated using a lower throughput, 96-well format (not shown). Dose-dependent activity was confirmed, and fold-changes in promoter activity between 1.9 and 3.5 were achieved.

**Figure 4:**
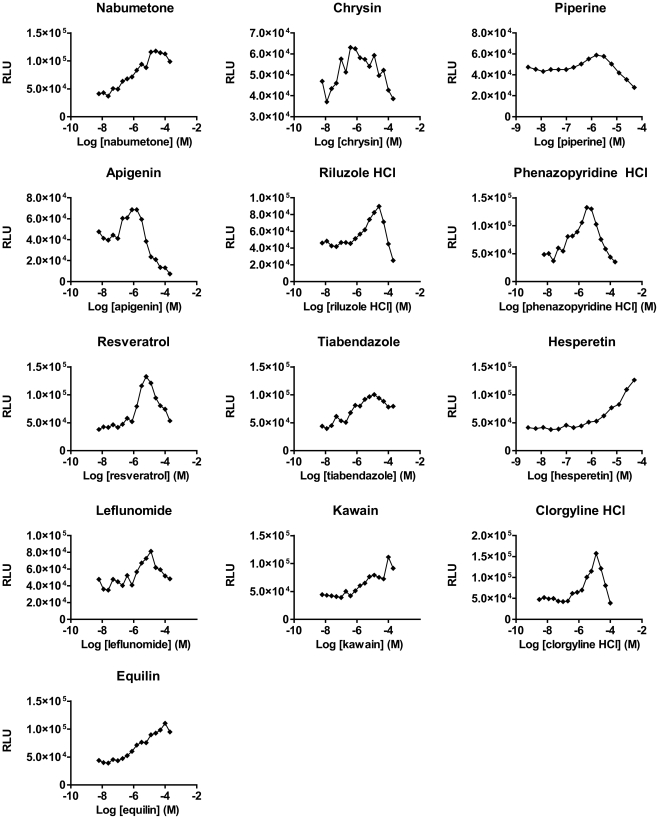
Dose optimisation: dose-response II. Dose-response curves were generated in 384-well format for the 14 confirmed hits using concentrations from 3.1 nM to 200 µM. HCl, hydrochloride. RLU, relative luminescence units.

For confirmed hits, stock solutions of higher concentration (100 or 200 mM) were obtained, and dose-response testing repeated using concentrations up to 200 µM (dose-response II; Fig. 4 and Table 3). Based on this, nabumetone, an FDA-approved drug that showed high fold-changes and a lack of cellular toxicity (indicated by a drop in luciferase activity at higher concentrations, e.g. piperine, Fig. 4) was selected for independent validation.

### Independent Validation

**Figure 5:**
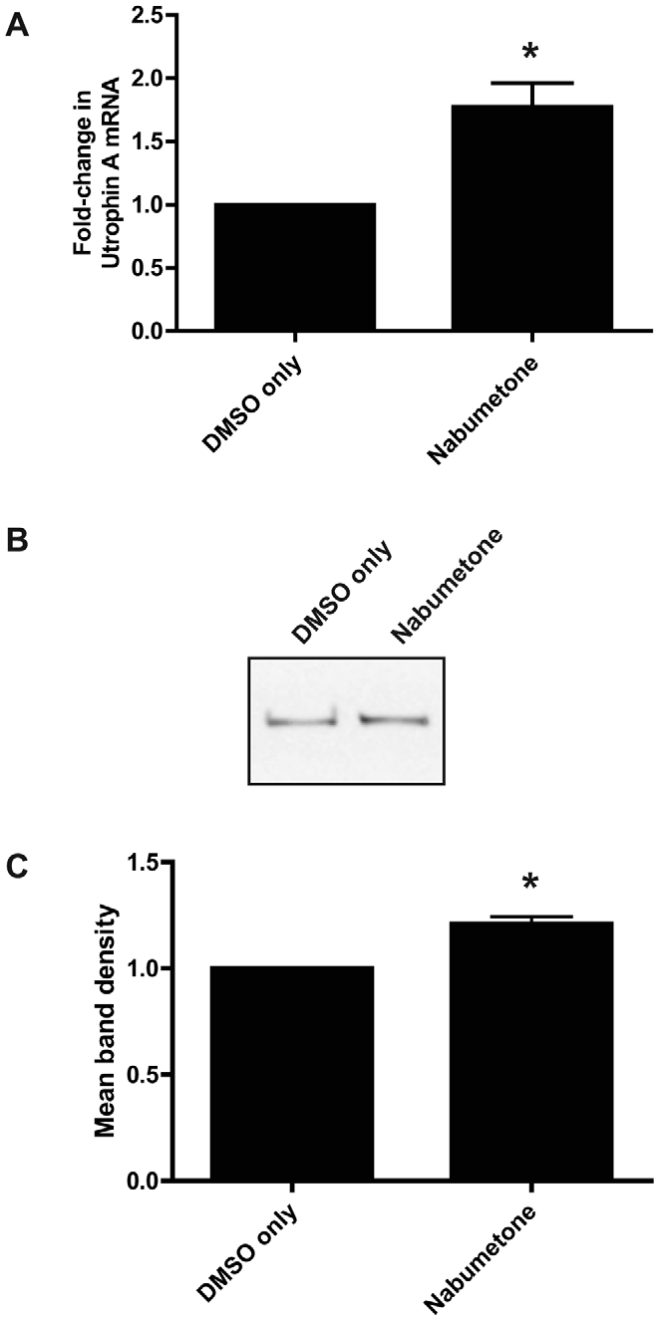
Independent validation of nabumetone. **A.** C2C12 cells were treated with nabumetone (25 µM) or DMSO only for 24 hours. Nabumetone produced a statistically significant, 1.8-fold increase in utrophin A mRNA. Bars represent means of four independent experiments ± standard error. * Different from DMSO only controls by Student's T test (p<0.05). **B.** Representative Western blot showing protein lysates from C2C12 cells treated with nabumetone or DMSO only for 4 days, probed for utrophin with the antibody mancho3. A single band was detected which migrated above the 250 kDa marker (markers not shown). **C.** Quantification of utrophin band densities from three independent Western blotting experiments, using ImageJ. Nabumetone treatment resulted in a 1.2-fold increase in utrophin protein. Bars represent means of 3 independent experiments, each consisting of 3 nabumetone-treated and 3 DMSO only control samples, ± standard error. * Different from DMSO only controls by Student's T test (p<0.05).

To confirm the effect of nabumetone on endogenous utrophin promoter activity, normal C2C12 cells were treated with nabumetone at its optimum concentration based on dose-response testing, and utrophin A mRNA levels measured using a TaqMan qRT-PCR assay. Treatment with nabumetone resulted in a statistically significant, 1.8-fold increase in endogenous utrophin A mRNA, compared to DMSO only controls (Fig. 5A).

As further validation, C2C12 cells were treated with nabumetone for 4 days, and utrophin protein levels were measured by Western blotting. As shown in Fig. 5B–C, nabumetone treatment resulted in a 1.2-fold increase in utrophin protein, confirming that the observed upregulation of utrophin mRNA led to an increase in protein levels.

## Discussion

In this study, we present a novel utrophin promoter activation assay, which we have used to screen a library of approved drugs and natural compounds. After initial screening, hit confirmation and independent validation, we have identified a lead compound, nabumetone, that is a potential therapeutic candidate for DMD.

The utrophin promoter activation assay performed well in tests of robustness, with a Z-factor of 0.6 and % CV under 10%. The number of hits as a percentage of the library (1.8%) was comparable to other published screens [36], [37], [38], suggesting that the assay was specific with a low number of false positives. Nevertheless, we had set the initial screen threshold of 20% upregulation low enough to avoid false negatives, in the expectation that some of the initial hits would be false positives due to ‘statistical noise’. Consistent with this, only 14 out of 20 initial hits (70%) were confirmed upon dose-response testing.

In screening assays that use luciferase as a reporter, false positives can arise from compounds that act as luciferase inhibitors. These compounds bind to and stabilise luciferase in cells, increasing its levels, and are then competed off by the substrate in the luciferase assay reagent, such that an artifactually high luciferase activity is produced [39]. Thus, it is important to independently confirm the effects of the hit compounds on the endogenous utrophin A promoter, mRNA and protein. We did this for one candidate, nabumetone, using a TaqMan qRT-PCR assay for utrophin A mRNA and Western blotting for utrophin protein. Validation of the remaining compounds is ongoing. However, definitive *in vitro* validation experiments are challenging, in part due to the differences in utrophin protein expression compared to the *in vivo* situation, where utrophin is enriched at specific locations, such as neuromuscular junctions [40], [41], [42], that do not exist in cultured cells. To move our findings closer to the clinic, it will be essential to determine the efficacy of the compounds *in vivo*, using animal models of DMD.

Previous studies suggest that an increase of approximately 2-fold in utrophin protein in muscle is sufficient for correction of the dystrophic phenotype in mice [18]. In our study, we identified several compounds that could upregulate the utrophin A promoter up to 3.5-fold. Independent validation of nabumetone showed that it could increase endogenous utrophin A mRNA levels approximately 2-fold, and increase utrophin protein by 1.2-fold. This is extremely promising given that even a very small upregulation of utrophin appears to have a beneficial effect in dystrophin deficient mice [18].

A variety of potential therapies for DMD are being investigated, and some have reached clinical trials (http://www.clinicaltrials.gov/). While this is greatly encouraging, there are still many obstacles to be overcome before all patients with DMD can be treated successfully. In many cases, problems of delivery, safety and large-scale, cost-effective manufacture have not yet been resolved. Some approaches, such as antisense oligonucleotide-mediated exon-skipping and nonsense codon suppression, are only applicable to subsets of patients with particular types of dystrophin mutations [43], [44]. Regulatory body approval may also be more complicated for new kinds of drug molecules such as proteins and oligonucleotides. For example, under current regulation, each of the potentially hundreds [43] of mutation-specific exon-skipping oligonucleotides would be treated as separate drugs [45]. It is also important to consider that, initially at least, combinations of treatments may be needed in order to achieve therapeutic efficacy. Therefore, the continuation of research along multiple therapeutic avenues, including utrophin upregulation, is of great importance to ensure the development of effective therapies for all patients with DMD.

There are a number of advantages to small molecule-mediated utrophin upregulation that make it both a strong candidate for DMD therapy and a complimentary approach to those discussed above. The introduction of dystrophin protein into the body of a DMD patient where it has never been expressed could provoke an immune reaction against the protein, which might be recognised as ‘non-self’. Utrophin is expressed in muscle in the foetus and at high levels in other tissues such as liver, lung and kidney throughout life in DMD patients (as well as healthy people) [9], [10], so increasing its production therapeutically in muscle would not risk inciting an immune response. Additionally, the utilisation of the endogenous utrophin gene provides an elegant solution to problems arising from the large size of the dystrophin coding sequence (14 kilobases) [46], which makes it difficult to incorporate into viral vectors, except in truncated form. Finally, the use of a traditional ‘drug-like’ small molecule, with favourable absorption, distribution, metabolism and excretion properties [47], [48], to upregulate utrophin offers obvious advantages in terms of delivery, stability and bioavailability.

Drug repositioning, the exploitation of existing drugs for new applications, is becoming an increasingly important part of research and development for the pharmaceutical industry [27], [28], [29]. Our approach of screening a library of regulatory body-approved drugs and natural compounds offers a distinct advantage in terms of the speed and efficiency of future therapy development. All the hits identified in our screen are compounds that have been shown to be safe in humans, and for which pharmacokinetic data is available. This eliminates a significant proportion of the time and expense required when developing a novel compound as a drug, and gives the potential for a rapid progression from the lab to the clinic.

To date, we have validated one drug, nabumetone, at the mRNA and protein level, in C2C12 cells. Nabumetone is a COX-1/COX-2 inhibitor that shows a preference for COX-2 inhibition *in vitro* [49]. It is used for the management of pain and inflammation in osteoarthritis and rheumatoid arthritis [50]. Nabumetone is generally well-tolerated by patients [50], and its anti-inflammatory activity might be beneficial in DMD, since inflammation is a component of the disease [6], [51], [52]. There is some evidence that the use of selective COX-2 inhibitors may increase the risk of adverse cardiovascular events, especially in patients who already have an increased risk [53], [54], [55]. This is less of a concern with COX-1/COX-2 inhibitors, possibly because COX-1 inhibition has an antiplatelet effect, which may protect against thrombotic events [56]. Nonetheless, because of the involvement of the heart in DMD pathology, the safety of nabumetone use in this group would need to be carefully evaluated.

In developing our assay, we used as positive controls a number of substances already known to upregulate utrophin: heregulin, TSA, okadaic acid and L-arginine. Of these, only L-arginine has been used in human beings, as a supplement. There are some safety concerns about its use, particularly at high dosages [57], [58]. Its use in DMD patients has not been investigated.

In terms of doses, it is not possible to directly compare *in vitro* and *in vivo* doses without considering pharmacokinetics; however, using a crude calculation based on an average total human body fluid volume of 42 l, the optimum dose for nabumetone determined in cell culture (25 µM) lies far below that used in human beings (equivalent to approximately 100–200 µM). As a comparison, L-arginine was effective in activating our utrophin upregulation assay at 2 mM, whereas doses used in humans would correspond roughly to 0.2–5 µM.

In our experiments, we observed a smaller increase in utrophin at the protein level than at the mRNA level. Although these experiments were done at different time points to allow for the expected slow synthesis of the large utrophin protein (approximately 400 kDa), this difference may also reflect the regulation of utrophin at the translational level. Indeed, it is known that utrophin expression is influenced by post-transcriptional mechanisms, acting via the 5′- and 3′-untranslated regions (UTRs) of the utrophin mRNA [59], [60], [61], [62]. It may be that by combining drugs that activate the utrophin promoter with therapies targeting points of post-transcriptional expression control, or therapeutic substances such as biglycan that promote localisation and stabilisation of utrophin at the sarcolemma [63], a far greater upregulation of utrophin can be achieved.

In conclusion, we have taken a novel approach to the problem of DMD therapy by screening existing drugs for utrophin promoter activation. Following the successful screening project and independent validation presented here, the lead compound nabumetone will be tested in preclinical trials for its ability to upregulate utrophin *in vivo* and improve the phenotype of dystrophic *mdx* mice. This venture offers great promise for the rapid development of an effective drug therapy for DMD. However, we caution that although nabumetone is an FDA-approved drug that is used safely in human beings, it will still be important to conduct thorough preclinical studies in animals, as well as clinical trials, to determine the safety and efficacy of its long-term use in DMD.

## Supporting Information

Figure S1 **Development of utrophin promoter activation assay.** **A.** A standard curve with increasing concentrations of recombinant luciferase was generated in the presence or absence of normal C2C12 muscle cells. The presence of C2C12 cells had no effect on luciferase activity, as tested by two-way ANOVA. Error bars represent standard deviation. RLU, relative luminescence units. **B.** C2C12utrn cells were treated with various concentrations of DMSO for 48 hours and their luciferase activity assayed. Luciferase activity declined above 0.2% DMSO but at 0.5% DMSO was still 75% that of cells without DMSO. Error bars represent standard deviation.
(TIF)

## References

[1] Hoffman EP, Brown RH, Kunkel LM (1987). Dystrophin: the protein product of the Duchenne muscular dystrophy locus.. Cell.

[2] Engel AG, Franzini-Armstrong C (2004). Myology: Basic and Clinical.

[3] Durbeej M, Campbell KP (2002). Muscular dystrophies involving the dystrophin-glycoprotein complex: an overview of current mouse models.. Current Opinion in Genetics & Development.

[4] Wrogemann K, Pena SDJ (1976). Mitochondrial Calcium Overload: A General Mechanism for Cell-Necrosis in Muscle Disease.. The Lancet.

[5] Bodensteiner JB, Engel AG (1978). Intracellular calcium accumulation in Duchenne dystrophy and other myopathies: a study of 567,000 muscle fibers in 114 biopsies.. Neurology.

[6] Arahata K, Engel AG (1984). Monoclonal antibody analysis of mononuclear cells in myopathies. I: Quantitation of subsets according to diagnosis and sites of accumulation and demonstration and counts of muscle fibers invaded by T cells.. Ann Neurol.

[7] Emery AE, Muntoni F (2003). Duchenne Muscular Dystrophy.

[8] Kohler M, Clarenbach CF, Bahler C, Brack T, Russi EW (2009). Disability and survival in Duchenne muscular dystrophy.. J Neurol Neurosurg Psychiatry.

[9] Khurana TS, Hoffman EP, Kunkel LM (1990). Identification of a chromosome 6-encoded dystrophin-related protein.. J Biol Chem.

[10] Love DR, Hill DF, Dickson G, Spurr NK, Byth BC (1989). An autosomal transcript in skeletal muscle with homology to dystrophin.. Nature.

[11] Matsumura K, Ervasti JM, Ohlendieck K, Kahl SD, Campbell KP (1992). Association of dystrophin-related protein with dystrophin-associated proteins in mdx mouse muscle.. Nature.

[12] Tinsley JM, Blake DJ, Roche A, Fairbrother U, Riss J (1992). Primary structure of dystrophin-related protein.. Nature.

[13] Koenig M, Monaco AP, Kunkel LM (1988). The complete sequence of dystrophin predicts a rod-shaped cytoskeletal protein.. Cell.

[14] Love DR, Morris GE, Ellis JM, Fairbrother U, Marsden RF (1991). Tissue distribution of the dystrophin-related gene product and expression in the mdx and dy mouse.. Proc Natl Acad Sci U S A.

[15] Burton EA, Tinsley JM, Holzfeind PJ, Rodrigues NR, Davies KE (1999). A second promoter provides an alternative target for therapeutic up-regulation of utrophin in Duchenne muscular dystrophy.. Proc Natl Acad Sci U S A.

[16] Weir AP, Burton EA, Harrod G, Davies KE (2002). A- and B-utrophin have different expression patterns and are differentially up-regulated in mdx muscle.. J Biol Chem.

[17] Gilbert R, Nalbantoglu J, Petrof BJ, Ebihara S, Guibinga GH (1999). Adenovirus-mediated utrophin gene transfer mitigates the dystrophic phenotype of mdx mouse muscles.. Hum Gene Ther.

[18] Tinsley J, Deconinck N, Fisher R, Kahn D, Phelps S (1998). Expression of full-length utrophin prevents muscular dystrophy in mdx mice.. Nat Med.

[19] Krag TO, Bogdanovich S, Jensen CJ, Fischer MD, Hansen-Schwartz J (2004). Heregulin ameliorates the dystrophic phenotype in mdx mice.. Proc Natl Acad Sci U S A.

[20] Barton ER, Morris L, Kawana M, Bish LT, Toursel T (2005). Systemic administration of L-arginine benefits mdx skeletal muscle function.. Muscle Nerve.

[21] Voisin V, Sebrie C, Matecki S, Yu H, Gillet B (2005). L-arginine improves dystrophic phenotype in mdx mice.. Neurobiol Dis.

[22] Sonnemann KJ, Heun-Johnson H, Turner AJ, Baltgalvis KA, Lowe DA (2009). Functional substitution by TAT-utrophin in dystrophin-deficient mice.. PLoS Med.

[23] Lu Y, Tian C, Danialou G, Gilbert R, Petrof BJ (2008). Targeting artificial transcription factors to the utrophin A promoter: effects on dystrophic pathology and muscle function.. J Biol Chem.

[24] DiMasi JA (2001). Risks in new drug development: approval success rates for investigational drugs.. Clin Pharmacol Ther.

[25] DiMasi JA, Hansen RW, Grabowski HG (2003). The price of innovation: new estimates of drug development costs.. Journal of Health Economics.

[26] Reichert JM (2003). Trends in development and approval times for new therapeutics in the United States.. Nat Rev Drug Discov.

[27] Ashburn TT, Thor KB (2004). Drug repositioning: identifying and developing new uses for existing drugs.. Nat Rev Drug Discov.

[28] Tobinick EL (2009). The value of drug repositioning in the current pharmaceutical market.. Drug News Perspect.

[29] Chong CR, Sullivan DJ (2007). New uses for old drugs.. Nature.

[30] Rothstein JD, Patel S, Regan MR, Haenggeli C, Huang YH (2005). [beta]-Lactam antibiotics offer neuroprotection by increasing glutamate transporter expression.. Nature.

[31] Baby SM, Bogdanovich S, Willmann G, Basu U, Lozynska O (2010). Differential Expression of Utrophin-A and -B Promoters in the Central Nervous System (CNS) of Normal and Dystrophic *mdx* Mice..

[32] Chaubourt E, Fossier P, Baux G, Leprince C, Israel M (1999). Nitric oxide and l-arginine cause an accumulation of utrophin at the sarcolemma: a possible compensation for dystrophin loss in Duchenne muscular dystrophy.. Neurobiol Dis.

[33] Rodova M, Brownback K, Werle MJ (2004). Okadaic acid augments utrophin in myogenic cells.. Neurosci Lett.

[34] Khurana TS, Rosmarin AG, Shang J, Krag TO, Das S (1999). Activation of utrophin promoter by heregulin via the ets-related transcription factor complex GA-binding protein alpha/beta.. Mol Biol Cell.

[35] Zhang JH, Chung TD, Oldenburg KR (1999). A Simple Statistical Parameter for Use in Evaluation and Validation of High Throughput Screening Assays.. J Biomol Screen.

[36] Ji DB, Zhu HB, Ye J, Li CL (2008). Establishment of a cell-based assay to screen regulators of the hypoxia-inducible factor-1-dependent vascular endothelial growth factor promoter.. Biol Pharm Bull.

[37] Jiang N, Ou-Yang KQ, Cai SX, Hu YH, Xu ZL (2005). Identification of human dopamine D1-like receptor agonist using a cell-based functional assay.. Acta Pharmacol Sin.

[38] Xu ZL, Gao H, Ou-Yang KQ, Cai SX, Hu YH (2004). Establishment of a cell-based assay to screen regulators for Klotho gene promoter.. Acta Pharmacol Sin.

[39] Auld DS, Thorne N, Nguyen D-T, Inglese J (2008). A Specific Mechanism for Nonspecific Activation in Reporter-Gene Assays.. ACS Chemical Biology.

[40] Khurana TS, Watkins SC, Chafey P, Chelly J, Tome FM (1991). Immunolocalization and developmental expression of dystrophin related protein in skeletal muscle.. Neuromuscul Disord.

[41] Nguyen TM, Ellis JM, Love DR, Davies KE, Gatter KC (1991). Localization of the DMDL gene-encoded dystrophin-related protein using a panel of nineteen monoclonal antibodies: presence at neuromuscular junctions, in the sarcolemma of dystrophic skeletal muscle, in vascular and other smooth muscles, and in proliferating brain cell lines.. J Cell Biol.

[42] Ohlendieck K, Ervasti JM, Matsumura K, Kahl SD, Leveille CJ (1991). Dystrophin-related protein is localized to neuromuscular junctions of adult skeletal muscle.. Neuron.

[43] Aartsma-Rus A, Ivo F, Jan V, Ieke G, Judith van D (2009). Theoretic applicability of antisense-mediated exon skipping for Duchenne muscular dystrophy mutations..

[44] Welch EM, Barton ER, Zhuo J, Tomizawa Y, Friesen WJ (2007). PTC124 targets genetic disorders caused by nonsense mutations.. Nature.

[45] Hoffman EP (2007). Skipping toward Personalized Molecular Medicine.

[46] Koenig M, Hoffman EP, Bertelson CJ, Monaco AP, Feener C (1987). Complete cloning of the Duchenne muscular dystrophy (DMD) cDNA and preliminary genomic organization of the DMD gene in normal and affected individuals.. Cell.

[47] Lipinski CA (2000). Drug-like properties and the causes of poor solubility and poor permeability.. J Pharmacol Toxicol Methods.

[48] Di L, Kerns EH (2003). Profiling drug-like properties in discovery research.. Current Opinion in Chemical Biology.

[49] Laneuville O, Breuer DK, Dewitt DL, Hla T, Funk CD (1994). Differential inhibition of human prostaglandin endoperoxide H synthases-1 and -2 by nonsteroidal anti-inflammatory drugs.. J Pharmacol Exp Ther.

[50] Hedner T, Samulesson O, Wahrborg P, Wadenvik H, Ung KA (2004). Nabumetone: therapeutic use and safety profile in the management of osteoarthritis and rheumatoid arthritis.. Drugs.

[51] McDouall RM, Dunn MJ, Dubowitz V (1990). Nature of the mononuclear infiltrate and the mechanism of muscle damage in juvenile dermatomyositis and Duchenne muscular dystrophy.. J Neurol Sci.

[52] Chen YW, Nagaraju K, Bakay M, McIntyre O, Rawat R (2005). Early onset of inflammation and later involvement of TGFbeta in Duchenne muscular dystrophy.. Neurology.

[53] Bombardier C, Laine L, Reicin A, Shapiro D, Burgos-Vargas R (2000). Comparison of upper gastrointestinal toxicity of rofecoxib and naproxen in patients with rheumatoid arthritis. VIGOR Study Group.. N Engl J Med.

[54] Bresalier RS, Sandler RS, Quan H, Bolognese JA, Oxenius B (2005). Cardiovascular Events Associated with Rofecoxib in a Colorectal Adenoma Chemoprevention Trial..

[55] Solomon SD, McMurray JJV, Pfeffer MA, Wittes J, Fowler R (2005). Cardiovascular Risk Associated with Celecoxib in a Clinical Trial for Colorectal Adenoma Prevention..

[56] FitzGerald GA, Oates JA, Hawiger J, Maas RL, Roberts LJ (1983). Endogenous biosynthesis of prostacyclin and thromboxane and platelet function during chronic administration of aspirin in man.. J Clin Invest.

[57] Schulman SP, Becker LC, Kass DA, Champion HC, Terrin ML (2006). L-Arginine Therapy in Acute Myocardial Infarction: The Vascular Interaction With Age in Myocardial Infarction (VINTAGE MI) Randomized Clinical Trial..

[58] U.S. National Library of Medicine NIoH (2010). Drugs & Supplements: L-arginine.. http://www.nlm.nih.gov/medlineplus/druginfo/natural/875.html.

[59] Gramolini AO, Belanger G, Jasmin BJ (2001). Distinct regions in the 3′ untranslated region are responsible for targeting and stabilizing utrophin transcripts in skeletal muscle cells.. J Cell Biol.

[60] Gramolini AO, Belanger G, Thompson JM, Chakkalakal JV, Jasmin BJ (2001). Increased expression of utrophin in a slow vs. a fast muscle involves posttranscriptional events.. Am J Physiol Cell Physiol.

[61] Miura P, Thompson J, Chakkalakal JV, Holcik M, Jasmin BJ (2005). The utrophin A 5′-untranslated region confers internal ribosome entry site-mediated translational control during regeneration of skeletal muscle fibers.. J Biol Chem.

[62] Rosenberg MI, Georges SA, Asawachaicharn A, Analau E, Tapscott SJ (2006). MyoD inhibits Fstl1 and Utrn expression by inducing transcription of miR-206.. J Cell Biol.

[63] Amenta AR, Yilmaz A, Bogdanovich S, McKechnie BA, Abedi M Biglycan recruits utrophin to the sarcolemma and counters dystrophic pathology in mdx mice..

