# Supplementary figures and images for: Drug Discovery for Duchenne Muscular Dystrophy via Utrophin Promoter Activation Screening
Source: PLoS One. 2011 Oct 20;6(10):e26169. doi: 10.1371/journal.pone.0026169 (PMC3197614; doi:10.1371/journal.pone.0026169)

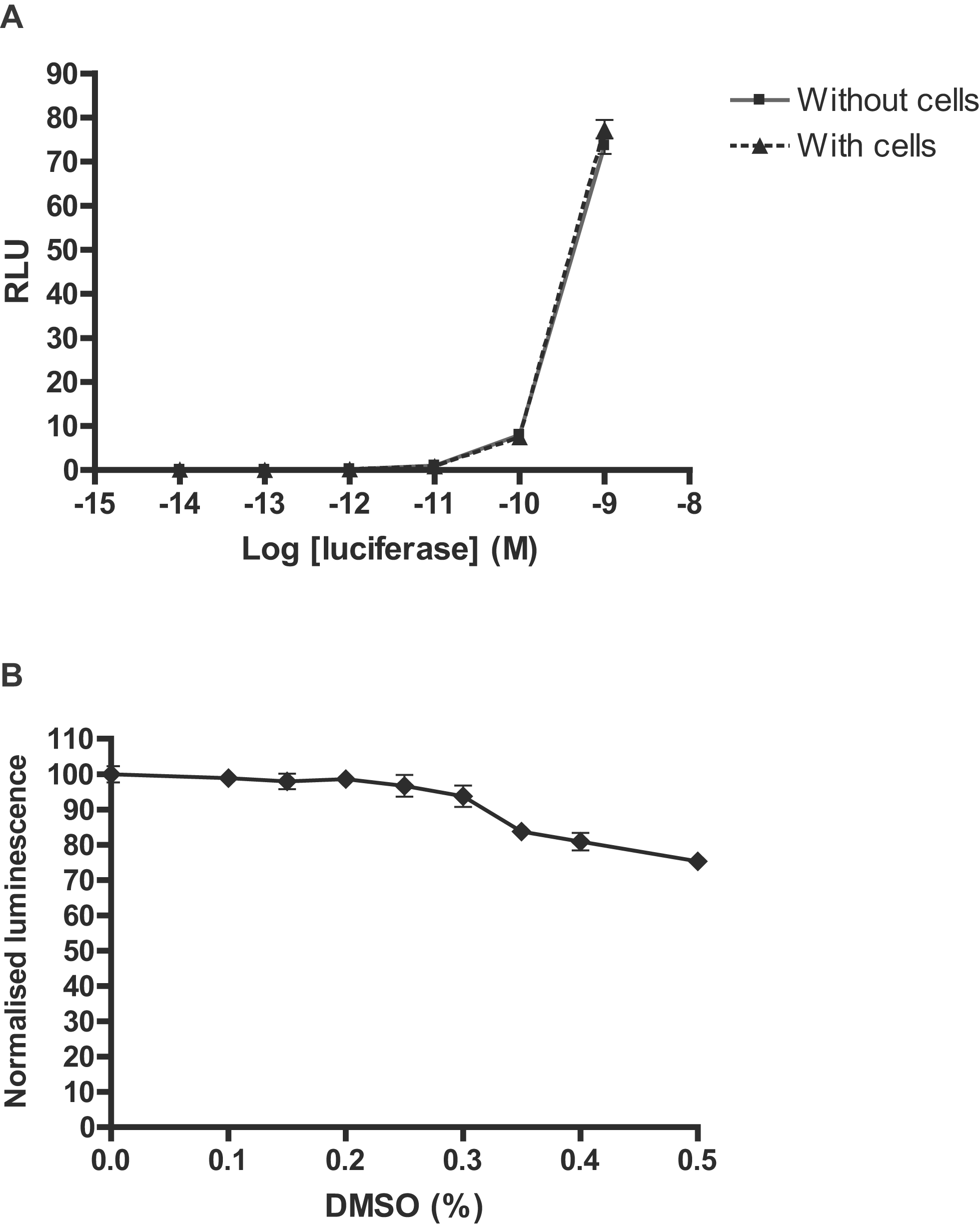

Supplement: Figure S1 — Development of utrophin promoter activation assay. A. A standard curve with increasing concentrations of recombinant luciferase was generated in the presence or absence of normal C2C12 muscle cells. The presence of C2C12 cells had no effect on luciferase activity, as tested by two-way ANOVA. Error bars represent standard deviation. RLU, relative luminescence units. B. C2C12utrn cells were treated with various concentrations of DMSO for 48 hours and their luciferase activity assayed. Luciferase activity declined above 0.2% DMSO but at 0.5% DMSO was still 75% that of cells without DMSO. Error bars represent standard deviation. (TIF) [file pone.0026169.s001.tif]
